# The Immunostimulatory Effect of MIL-101(Al)-NH_2_ In Vivo and Its Potential to Overcome Bacterial Resistance to Penicillin Enhanced by Hypericin-Induced Photodynamic Therapy

**DOI:** 10.3390/ijms262311681

**Published:** 2025-12-02

**Authors:** Mariana Máčajová, Ľuboš Ambro, Majlinda Meta, Ľuboš Zauška, Terézia Gulyásová, Boris Bilčík, Ivan Čavarga, Gabriela Zelenková, Erik Sedlák, Miroslav Almáši, Veronika Huntošová

**Affiliations:** 1 Institute of Animal Biochemistry and Genetics, Centre of Biosciences, Slovak Academy of Sciences, Dúbravská Cesta 9, SK-840 05 Bratislava, Slovakia; mariana.macajova@savba.sk (M.M.); majlinda.meta@savba.sk (M.M.); boris.bilcik@savba.sk (B.B.); ivan.cavarga@savba.sk (I.Č.); 2 Center for Interdisciplinary Biosciences, Technology and Innovation Park, Pavol Jozef Šafárik University in Košice, Jesenná 5, SK-041 54 Košice, Slovakia; lubos.ambro@upjs.sk (Ľ.A.); terezia.gulyasova@student.upjs.sk (T.G.); erik.sedlak@upjs.sk (E.S.); 3 Department of Inorganic Chemistry, Faculty of Science, Pavol Jozef Šafárik University in Košice, Moyzesova 11, SK-041 54 Košice, Slovakia; lubos.zauska@upjs.sk; 4 Department of Biophysics, Faculty of Science, Pavol Jozef Šafárik University in Košice, Jesenná 5, SK-041 54 Košice, Slovakia; 5 Department of Chemistry, Faculty of Science, University of Ostrava, 30, Dubna 22, CZ-701 03 Ostrava, Czech Republic; gabriela.zelenkova@osu.cz; 6 Department of Biochemistry, Faculty of Science, Pavol Jozef Šafárik University in Košice, Moyzesova 11, SK-041 54 Košice, Slovakia

**Keywords:** metal-organic frameworks, MIL-101(Al)-NH_2_, immune response, antibacterial photodynamic therapy, bacterial resistance, penicillin, hypericin, chorioallantoic membrane model

## Abstract

The increasing prevalence of multidrug-resistant bacteria necessitates alternative therapeutic strategies that combine antimicrobial efficacy with immunomodulatory properties. Here, we report the immunostimulatory activity and antibacterial potential of the amino-functionalized metal–organic framework MIL-101(Al)-NH_2_ as a carrier for penicillin (PEN) and hypericin (Hyp), a photodynamically active compound. Structural and physicochemical characterization confirmed successful encapsulation of PEN, Hyp, and their combination within MIL-101(Al)-NH_2_, with distinct effects on porosity, release kinetics, and thermal stability. Drug release studies revealed rapid Hyp liberation triggered by serum components, whereas PEN exhibited a biphasic, diffusion-controlled profile. Using a quail chorioallantoic membrane (CAM) model, we demonstrated that MIL-101(Al)-NH_2_ enhances interferon-α expression, indicating intrinsic immunostimulatory activity, and that Hyp-loaded systems promote angiogenic responses. In a bacterial infection CAM model, MIL-101(Al)-NH_2_ carriers loaded with Hyp or Hyp/PEN induced immunomodulatory changes and, upon photodynamic activation, inhibited bacterial growth. While Gram-negative *Escherichia coli* remained resistant, Gram-positive *Staphylococcus epidermidis* was effectively suppressed by photodynamic therapy (PDT), and Hyp/PEN co-delivery overcame bacterial resistance to PEN. These results highlight MIL-101(Al)-NH_2_ as a multifunctional nanoplatform with immunostimulatory capacity and PDT-enhanced antibacterial activity, offering a promising strategy to combat antibiotic resistance and infections associated with medical implants.

## 1. Introduction

Wound healing is a complex process in which multiple reparative mechanisms take place at different levels. If one of these mechanisms is disturbed, this can lead to the development of non-healing chronic ulcers. Healing occurs in three basic, progressive processes in the form of inflammation, proliferation and remodeling. The microbial load of wounds has a fundamental influence on the healing process and the closure of the defect. The interaction of microbial pathogens in the wound environment also occurs in successive phases such as contamination, critical colonization and infection [1,2]. These complex processes require a multimodal approach to wound management that goes beyond current anti-inflammatory and antimicrobial therapy. Wound healing is made more difficult by the multiresistance of the microbial pathogen and endogenous factors of the organism, such as atherosclerosis, diabetes and immunodeficiency [2,3].

Decreasing the effectiveness of conventional therapeutic procedures has stimulated an increased effort to discover new classes of functional materials, among which metal–organic frameworks (MOFs) and their derivatives attract particular attention [4]. MOFs are crystalline porous coordination polymers composed of metal nodes or clusters interconnected by multidentate organic linkers. Their highly ordered structures combine the advantages of inorganic and organic components, resulting in exceptionally high surface areas, tunable pore sizes, and versatile chemical functionalities. These properties make MOFs suitable for applications in adsorption [5,6], separation [7], energy storage [8,9,10], environmental remediation [11,12], and drug delivery [13,14]. Mesoporous aluminum-based MOFs, such as MIL-101(Al) and its analogues, are especially attractive because they can adsorb and encapsulate a wide spectrum of molecules while simultaneously catalyzing a broad range of reactions. Moreover, they can be easily functionalized through post-synthetic modifications or converted into oxide and carbonaceous materials with intriguing physicochemical properties [15]. In particular, amino-functionalized MOFs, where amino groups are incorporated into the linker, display a characteristic fluorescence response upon excitation with UVA radiation and blue light. The fluorescence intensity is strongly dependent on the analyte concentration, providing an opportunity for sensitive detection and bioimaging applications [16,17].

The properties of MIL-101(Al)-NH_2_ in interaction with the biological environment have not been sufficiently studied. An interesting immunostimulatory effect of MOFs and also an antimicrobial potential, which may be linked to the presence of metal in the molecule, are assumed [4,18,19,20].

MOFs have interesting properties that make them lucrative for various biological applications. Their large surface area and tailored porosity allow biologically relevant and specific functionalization, enabling their use in cell-targeted treatment [21,22]. In addition, the photocatalytic activity enables the generation of reactive oxygen species (ROS), which form the basis of PDT [23]. The adsorption of photosensitizers in the pores makes MOFs valuable agents for antibacterial and antimicrobial treatment [24,25].

Their advantages over antibiotics are their high biocompatibility and degradability, their non-specific nature and broad spectrum of use, their good efficacy in killing and their low bacterial resistance [26,27]. In addition, antibiotics themselves can also be encapsulated in MOFs, thereby circumventing the problem of resistance and increasing their uptake by the cell [28].

Several studies have shown that combining MOFs with simple antibiotics can reduce bacterial resistance, including in strains such as *E. coli* and *S. aureus* [29,30]. Penicillin G is a well-characterized and widely used β-lactam antibiotic that serves as an appropriate probe molecule for systems of this type. In addition, its good solubility and chemical stability in aqueous media make it suitable for kinetic release studies [31].

Moreover, penicillin G may be considered the prototypical β-lactam antibiotic, from which all clinically used semisynthetic penicillins were derived. Using it as a reference compound allows us to establish a proof-of-concept for drug encapsulation and release behavior from MIL-101(Al)–NH_2_, which can then be extended to more advanced analogues in future work.

The antibacterial activity of MOFs is related to the release and degradation of active metallic and organic components [32]. Both Gram-positive and Gram-negative bacteria are susceptible to the metal ions present in MOFs. Ag^+^, Al^3+^, Cu^2+^ and Zn^2+^ have been shown to interfere with the metabolic activity of bacteria and are therefore suitable for the development of bactericidal MOFs [4,32]. The released metals can come into direct contact with the bacterial cell. This leads to the production of ROS, oxidative stress and destruction of the cell wall, which is accompanied with a massive leakage of cell contents, DNA damage, loss of proton gradient, and inactivation of proteins in bacterial cells [33,34]. Antibacterial activity of MOFs can be significantly increased by organic ligands of which (antibacterial) properties can be properly designed. Simultaneous release of organic compounds and metal ions can thus lead to synergetic antibacterial effect of MOFs [33,35,36].

We have previously shown that MIL-101(Al)-NH_2_ adsorbs hypericin (Hyp), a photodynamically active molecule that can generate highly reactive ROS, singlet oxygen (^1^O_2_), after irradiation with 590 nm [37]. This complex has proven to be very effective in the treatment of cancer, viruses and bacteria. Chi and Du have shown that MIL-101(Al)-NH_2_ can be modified with AgI nanoparticles and exhibits selective antibacterial photoactivity after stimulation with 420 nm [38]. This gave rise to the idea of the present work, which is based on potentially effect of Hyp and penicillin (PEN) by their combination and overcome bacterial resistance to PEN by photodynamic treatment (PDT) at 405 and 590 nm. Differences in antibacterial treatment between Gram-positive and Gram-negative bacteria were found. In addition, an in vivo model of bacterial infection of the chorioallantoic membrane (CAM) of quails was created as a valuable model for the evaluation of antibacterial treatments and assessed by PDT at 405 nm using molecular biological and histological methods.

When combined with Hyp and PEN, the MIL-101(Al)-NH_2_ system exhibited synergistic antibacterial and immune-enhancing effects against *E. coli*. In the quail CAM model, this composite not only improved photodynamic inactivation of bacteria but also stimulated local immune responses, enhancing vascular activation and inflammatory signaling. These results highlight the potential of MOF-based platforms such as MIL-101(Al)-NH_2_ to integrate antibacterial PDT with host immunomodulation for effective treatment of Gram-negative infections.

## 2. Results

### 2.1. Characterization of MIL-101(Al)-NH_2_ and MIL-101(Al)-NH_2_ Loaded with PEN, Hyp and Their Combination

MIL-101(Al)-NH_2_ and its drug-loaded composites with PEN and Hyp were characterized by FT-IR spectroscopy, powder X-ray diffraction (PXRD), nitrogen adsorption/desorption, and thermogravimetric (TGA) measurements to evaluate their functional groups, crystallinity, textural parameters, and thermal behavior, including drug content.

**Figure 1:**
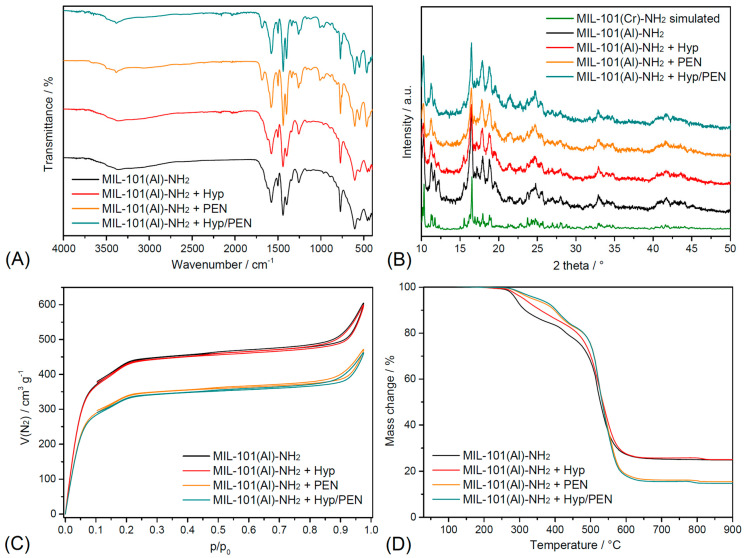
Structural and physicochemical characterization of pristine MIL-101(Al)-NH_2_ and its drug-loaded composites (Hyp, PEN, Hyp/PEN): (**A**) FTIR spectra, (**B**) PXRD patterns, (**C**) N_2_ adsorption–desorption isotherms at 77 K, and (**D**) thermogravimetric profiles.

The FT-IR spectra of MIL-101(Al)–NH_2_ and its drug-loaded forms are shown in Figure 1A. For the pristine MOF, characteristic absorption bands of the 2-aminoterephthalate linker are observed at 3478 and 3362 cm^−1^, corresponding to N–H stretching vibrations. The scissoring *δ*(N–H) mode appears at 1620 cm^−1^, while the asymmetric and symmetric stretching vibrations of the carboxylate groups are located at 1576 and 1439 cm^−1^, respectively. The C–O stretching is detected at 1255 cm^−1^, and the *δ*(COO^−^) vibration at 770 cm^−1^ [39]. Finally, the Al–O stretching band observed around 604 cm^−1^ confirms coordination of the carboxylate linkers to Al^3+^ nodes [40]. After drug loading, the IR spectra of the composites remain largely similar to that of the parent MOF, demonstrating that the framework structure is preserved. In the case of the PEN-loaded sample, a new absorption band is observed at 1684 cm^−1^, corresponding to the C=O stretching vibration of PEN, and the N–H stretching region shows an intensified band at 3379 cm^−1^, further supporting the presence of PEN in the framework. For the Hyp-loaded material, no additional strong absorption bands characteristic of Hyp can be detected. For the dual system (Hyp/PEN), the spectra are dominated by the characteristic contributions of PEN, namely the C=O stretching and the intensified N–H band, while no distinct vibrations of Hyp can be identified due to its low loading (0.2 mg per 1 g of support).

PXRD analysis was employed to verify the structure of MIL-101(Al)–NH_2_ and to assess the structural integrity of the framework after drug encapsulation (Figure 1B). The experimental diffraction patterns of the pristine MIL-101(Al)–NH_2_ and the drug-loaded composites were compared with the simulated pattern of MIL-101(Cr)–NH_2_ calculated from single-crystal data [41]. In all cases, the characteristic reflections of the MIL-101 framework are clearly visible, confirming that the Al analogue was successfully obtained and that the framework topology was preserved after loading with the active compounds. The PXRD profiles of MIL-101(Al)–NH_2_ before and after Hyp incorporation remain highly similar, demonstrating that the host lattice is stable upon drug uptake. In the case of PEN-loaded MIL-101(Al)–NH_2_, as well as the dual system containing both PEN and Hyp, the reflections of the MIL-101 framework are still maintained. Importantly, no additional diffraction peaks attributable to crystalline PEN were detected. This observation suggests that PEN is stored within the pores in an amorphous state. This assumption is further supported by the release studies presented in a later section, where PEN exhibited faster release kinetics, in agreement with the fact that amorphous pharmaceuticals dissolve more readily due to the absence of an energy barrier associated with crystal lattice disruption [42,43]. The PXRD results confirm that the MIL-101(Al)–NH_2_ framework retains its structural integrity after the encapsulation of Hyp, PEN, and their combination.

The N_2_ adsorption/desorption isotherms of pristine MIL-101(Al)–NH_2_ and its drug-loaded composites, measured at −196 °C, are shown in Figure 1C. All samples exhibit type *IV* isotherms with a pronounced uptake at low relative pressures (*p*/*p*_0_ < 0.1), confirming the presence of micropores, followed by a hysteresis loop associated with capillary condensation in the mesopores. Pristine MIL-101(Al)–NH_2_ displays a high BET surface area (*S_BET_* = 1570 m^2^·g^−1^) and a total pore volume (*V_p_*) of 0.92 cm^3^·g^−1^, with an average pore diameter (*d*) of 2.94 nm. Upon loading with hypericin, these values decrease only slightly (*S_BET_* = 1500 m^2^·g^−1^, *V_p_* = 0.91 cm^3^·g^−1^, *d* = 2.93 nm), indicating that the incorporation of a small amount of Hyp molecules leads to only partial pore occupation, while the overall mesoporous structure is preserved. In contrast, PEN-loaded MIL-101(Al)–NH_2_ exhibits a more significant reduction in textural properties, with *S_BET_* decreasing to 1190 m^2^·g^−1^ and *V_p_* to 0.70 cm^3^·g^−1^, accompanied by a contraction of the average pore diameter to 2.24 nm. This pronounced decrease confirms that PEN molecules are effectively accommodated within the pore system, resulting in partial pore blocking and reduced accessible surface area. The most pronounced changes are observed for the dual drug system (MIL-101(Al)–NH_2_ + Hyp/PEN), where *S_BET_* and *V_p_* further decrease to 1140 m^2^·g^−1^ and 0.68 cm^3^·g^−1^, respectively, with an average pore diameter of 2.22 nm. The isotherm profile remains of type *IV*, however, the diminished nitrogen uptake at both low and high relative pressures demonstrates the combined effect of Hyp and PEN molecules filling the micro- and mesopores. Overall, these results confirm the successful encapsulation of the bioactive compounds into MIL-101(Al)–NH_2_, with Hyp producing only a minor reduction in porosity, whereas PEN and especially the PEN/Hyp combination strongly influence the accessible surface area and pore volume while preserving the structural integrity of the MOF.

Thermogravimetric analysis (Figure 1D) was employed to evaluate the thermal stability of pristine MIL-101(Al)-NH_2_ and its drug-loaded composites. All TGA curves exhibited an initial weight loss up to approximately 10% in the temperature range of 30–80 °C, which can be attributed to the release of physisorbed water or residual methanol molecules, solvents used in the impregnation process. All the samples displayed comparable thermal stability up to 120 °C, above which the onset of decomposition was observed. The weight changes associated with the desolvation process were normalized [44] and considered in the evaluation of the decomposition of the framework and the encapsulated drugs, as well as the final residual masses. The thermal decomposition of pristine MIL-101(Al)-NH_2_ [Al_3_O(OH)(BDC-NH_2_)_3_] proceeds in three distinct steps within the temperature range of 120–620 °C, while at higher temperatures the formation of a stable residue corresponding to Al_2_O_3_ is observed. The calculated weight loss associated with framework combustion is 73.6 wt%, which is in good agreement with the experimental value of 74.9 wt%. The final residue amount of 25.1 wt% is consistent with the theoretical Al_2_O_3_ fraction (26.4 wt%) expected from the MOF composition. Drug-loaded materials with PEN showed additional mass losses in the 120–620 °C range, attributable to the decomposition of PEN overlapping with framework combustion. Consequently, their residual fractions decreased to 15.5 wt% for MIL-101(Al)-NH_2_ + PEN and 14.8 wt% for MIL-101(Al)-NH_2_ + Hyp/PEN, which is in line with the theoretical reduction expected for a 10 wt% PEN content (100 mg PEN per 1 g of MIL-101(Al)-NH_2_). In contrast, MIL-101(Al)-NH_2_ + Hyp displayed nearly identical thermal behavior to the pristine material, consistent with the negligible hypericin loading (0.2 mg per 1 g of MIL-101(Al)-NH_2_). However, the shape of the TG curve differs from the parent framework, since the two-step decomposition observed for pristine MIL-101(Al)-NH_2_ in the temperature range of 120–400 °C merges into a single degradation step for MIL-101(Al)-NH_2_ + Hyp, indicating a partial modification of the combustion pathway upon Hyp incorporation.

### 2.2. Drug Encapsulation Efficiency

The efficiency of drug encapsulation was evaluated indirectly based on changes in the total pore volume (*V_p_*) per gram of MIL-101(Al)–NH_2_, derived from nitrogen adsorption data. The calculated pore-filling fractions, determined from the relative decrease in *V_p_*, were 1.1% for Hyp [(0.92–0.91)/0.92], 23.9% for PEN [(0.92–0.70)/0.92], and 26.1% for the PEN/Hyp system [(0.92–0.68)/0.92]. To assess whether the available pore volume could accommodate the added drug mass, the minimum required solid density (*ρ_req_ = m*/*ΔV_p_*) was calculated for each system. The resulting values were 0.02 g·cm^−3^ for Hyp, 0.46 g·cm^−3^ for PEN, and 0.42 g·cm^−3^ for the PEN/Hyp combination. Given that typical organic solids have densities ranging from 1.0 to 1.6 g·cm^−3^, the calculated *ρ_req_* values are well within this range. This indicates that the observed reductions in pore volume are consistent with full retention of the impregnated drug mass. Consequently, the encapsulation efficiency (EE%) can be considered effectively 100% for all three systems under the employed conditions.

### 2.3. Drug Release Kinetics Study of MIL-101(Al)-NH_2_ Loaded with PEN, Hyp and Their Combination

**Figure 2:**
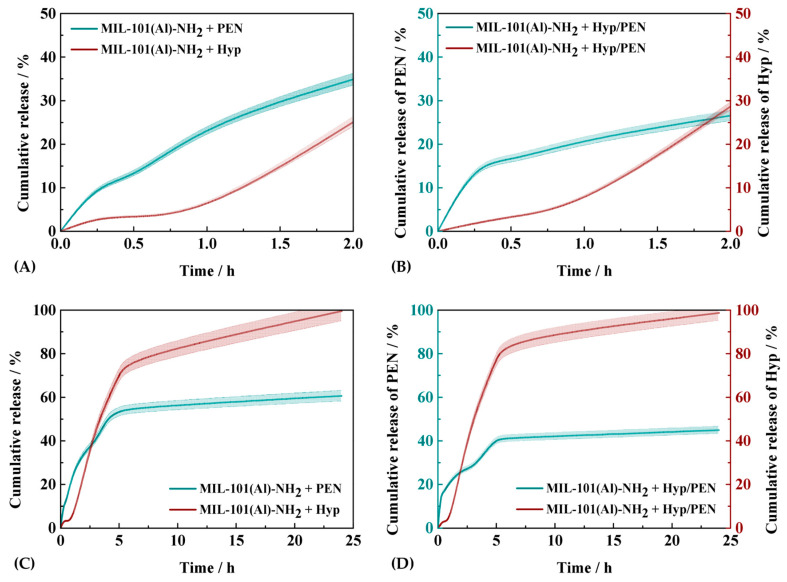
Hyp, PEN and Hyp/PEN release curves from MIL-101(Al)-NH_2_. FBS (nominal value of 10%) was added into the solutions after 1 h of measurement. Obtained dependences of the release of Hyp and PEN clearly indicate an increased dissociation of Hyp upon FBS addition suggesting Hyp redistribution into FBS. (**A**) Hyp and PEN release kinetics detected in first 2 h and (**C**) for 24 h. (**B**) Hyp and PEN release kinetics from Hyp/PEN loaded MIL-101(Al)-NH_2_ detected in first 2 h and (**D**) for 24 h.

The release behavior of Hyp, PEN, and their combinations from the MIL-101(Al)-NH_2_ carrier into physiological saline at *pH* 7.4, followed by the addition of fetal bovine serum (FBS), mimicked the experimental procedure of particle administration into biological cultures/models, where the particles were pre-dissolved in aqueous solutions before administration. During the first hour, the drugs and their combination were released exclusively into the saline solution. As shown in Figure 2A, Hyp was released only in trace amounts, with 3.2% released from MIL-101(Al)-NH_2_ + Hyp, and 2.8% from MIL-101(Al)-NH_2_ + Hyp/PEN, as illustrated in Figure 2B. In contrast, PEN exhibited markedly higher release, reaching 24.8% from MIL-101(Al)-NH_2_ + PEN and 21.2% from MIL-101(Al)-NH_2_ + Hyp/PEN, which points to the dilution of PEN from the external surface or interparticle void. Since the same amounts of drugs were encapsulated in the respective samples, these results indicate the absence of competitive release, with both drugs being released simultaneously. Upon the addition of FBS, a sharp increase in the cumulative release of Hyp was observed, with 25.8% released from MIL-101(Al)-NH_2_ + Hyp and 28.7% from MIL-101(Al)-NH_2_ + Hyp/PEN at the second hour of the experiment. In contrast, PEN continued to exhibit a more gradual release profile, with 34.6% released from MIL-101(Al)-NH_2_ + PEN and 26.6% from MIL-101(Al)-NH_2_ + Hyp/PEN, indicating that FBS had only a minor effect on its solubility and release kinetics. Figure 2C,D display the characteristic release profiles of drugs from MIL-101(Al)-NH_2_ [37,45,46]. For Hyp, the cumulative release from MIL-101(Al)-NH_2_ + Hyp followed a rapid and continuous trend, with 78.5% released within 6 h and nearly complete release (~100%) achieved by 24 h. This rapid behavior can be attributed to the high solubility of Hyp in FBS, and low interaction with the host matrix [47,48]. Although Hyp is, in principle, capable of forming hydrogen bonds, π–π stacking, and H–π interactions with the MIL-101(Al)–NH_2_ framework, the experimental evidence indicates only limited retention under the studied conditions. This conclusion is supported by its very low loading (0.2 mg per g of MOF), minimal changes in surface area and pore volume, and the absence of detectable shifts in PXRD or IR spectra (Section 2.1). Furthermore, the rapid and nearly complete release of Hyp upon addition of FBS suggests weak overall confinement, with non-covalent interactions contributing minimally to its retention within the MOF. This behavior implies that Hyp likely forms stronger interactions with serum proteins than with the MOF itself, favoring its desorption and solubilization in the biological medium. From the structural point of view, although MIL-101(Al)–NH_2_ contains two types of internal cages with different diameters (approximately 29 and 34 Å), both are accessible through pore windows of similar aperture size (1.2–1.6 nm), and thus cannot be regarded as separate microporous and mesoporous domains in the conventional sense. Given that both PEN and Hyp are significantly smaller than the pore windows, steric exclusion from either cage type is unlikely. Therefore, the observed differences in release profiles between PEN and Hyp are more plausibly attributed to the higher loading and stronger hydrogen-bonding interactions of PEN with the framework, rather than to diffusion limitations within smaller pores. This interpretation is further supported by the minimal structural changes and full release of Hyp in the presence of serum proteins.

A similar release pattern was observed for MIL-101(Al)-NH_2_ + Hyp/PEN, with 87% released within 6 h and nearly complete release by 24 h. In contrast, PEN demonstrated a distinctly different, biphasic release profile. The first release stage occurred within the initial 3 h, during which 39.6% was released from MIL-101(Al)-NH_2_ + PEN and 27.8% from MIL-101(Al)-NH_2_ + Hyp/PEN. The second stage extended to 6 h, reaching 55.5% and 41.3% cumulative release, respectively. This two-step profile suggests that PEN molecules occupy both types of cage pores within MIL-101(Al)-NH_2_ [49], whereas Hyp is confined to a single pore type. After 24 h, the cumulative release of PEN reached 60.6% and 45% for MIL-101(Al)-NH_2_ + PEN and MIL-101(Al)-NH_2_ + Hyp/PEN, respectively. The lower cumulative release compared to Hyp highlights the likely presence of hydrogen bonding interactions between the –COOH groups of PEN and the –NH_2_ functionalities of the MIL-101(Al)-NH_2_ framework. The drug release kinetics of MIL-101(Al)–NH_2_ carriers containing different drug loadings (PEN, Hyp, and their combinations) were analyzed using three mathematical models: first-order, Higuchi, and Korsmeyer–Peppas, despite the use of a porous carrier material. A non-standard modelling strategy was adopted that included segmented fitting of individual release stages, reflecting the distinct physicochemical behaviors of the two drugs. Hyp was loaded in small quantities, exhibited weak interactions with the framework, and was released rapidly. Accordingly, the first-order model was applied in the 1–24 h interval, since the other models provided poor correlation coefficients and were not suitable for this system.

In contrast, PEN was loaded at higher concentrations and formed hydrogen bonds with surface amine groups of the MIL-101(Al)–NH_2_ framework. Therefore, its release was analyzed in two segments: the initial (0–3 h) and secondary (3–6 h) stages were fitted with the Korsmeyer–Peppas model to capture the diffusion-controlled phase, while the first-order model was used for the overall 0–24 h interval to enable cross-sample comparison of release kinetics. This segmented modelling approach allowed differentiation between distinct release mechanisms: diffusion-dominated (Fickian to anomalous) transport for PEN and relaxation- or erosion-driven (super case-II) release for Hyp. It should also be noted that some kinetic rate constants exceeded 100%·g^−1^; these values are empirical extrapolations representing the theoretical maximum release predicted at infinite time.

The release of PEN from MIL-101(Al)–NH_2_ + PEN was well described by a first-order model over the entire time range (*R*^2^ = 0.9862; *Q*_∞_ = 55.9 ± 3.8%; *k*_1_ = 0.55 ± 0.08% h^−1^). A segmented analysis revealed a two-step release: within 0–3 h, the Korsmeyer–Peppas model fitted excellently (*R*^2^ = 0.9849; *k_kp_* = 22.1 ± 1.2% h^−n^; *n* = 0.60 ± 0.07), indicating anomalous (non-Fickian) diffusion, whereas for 3–6 h, the same model fitted less satisfactorily (*R*^2^ = 0.8354; *k_kp_* = 24.6 ± 4.4% h^−n^; *n* = 0.47 ± 0.11), consistent with Fickian diffusion. The Higuchi model, applied over 0–6 h, also showed an excellent correlation (*R*^2^ = 0.9881; *k_H_* = 22.8 ± 1.85% h^−1/2^), confirming that diffusion-controlled release dominated throughout the process.

The release of Hyp from MIL-101(Al)–NH_2_ + Hyp displayed a single-stage profile, beginning approximately 1 h after exposure to FBS. This delay corresponds to the onset of Hyp monomerization, as Hyp is poorly soluble in aqueous media but becomes solubilized in FBS through protein-mediated monomerization. Consequently, modelling was performed only for the 1–24 h interval, where the first-order model provided the best fit (*R*^2^ = 0.8956; *Q*_∞_ = 104.4 ± 11.2%; *k*_1_ = 0.22 ± 0.05% h^−1^), indicating diffusion-controlled release governed by desorption from internal pores once the drug–carrier system becomes wetted and stabilized in the serum environment.

For the MIL-101(Al)–NH_2_ + Hyp/PEN sample, the release of PEN showed a two-stage profile similar to the PEN-only system. Across the full 0–24 h interval, the data were best fitted by the Higuchi model (*R*^2^ = 0.9693; *k_H_* = 18.8 ± 1.3% h^−1/2^), tailed by the first-order model (*R*^2^ = 0.9861; *Q*_∞_ = 59.61 ± 2.6%; *k*_1_ = 0.55 ± 0.02% h^−1^), confirming a diffusion-controlled mechanism. A detailed segmented evaluation revealed that during the initial stage (0–3 h), the Korsmeyer–Peppas model gave an excellent fit (*R*^2^ = 0.9972; *k* = 21.2 ± 0.5% h^−n^; *n* = 0.25 ± 0.03), indicating quasi-Fickian diffusion with limited surface interaction. In the second stage (3–6 h), the same model fitted reasonably well (*R*^2^ = 0.9083; *k* = 14.8 ± 2.4% h^−n^; *n* = 0.60 ± 0.10), suggesting a transition to anomalous diffusion as the release slowed toward equilibrium.

The release behavior of Hyp from the MIL-101(Al)–NH_2_ + Hyp/PEN sample followed the same trend as observed for the Hyp-only system, showing a single-stage exponential release starting after approximately 1 h, due to Hyp monomerization in FBS. The first-order model again provided the best fit over the 1–24 h interval (*R*^2^ = 0.8956; *Q*_∞_ = 104.4 ± 11.2%; *k*_1_ = 0.22 ± 0.05% h^−1^), consistent with a diffusion-controlled exponential release of the monomeric species stabilized by serum proteins.

### 2.4. Immunostimulatory Effect of MIL-101(Al)-NH_2_ and MIL-101(Al)-NH_2_ Loaded with PEN, Hyp and Their Combination In Vivo

#### 2.4.1. Interaction of MIL-101(Al)-NH_2_ and MIL-101(Al)-NH_2_ Loaded with PEN, Hyp and Their Combination with CAM of Quail Embryo

The fluorescence pharmacokinetics and biocompatibility of the prepared MOFs were investigated in quail embryos. The aim of this study was to determine the amount of MOFs that remaining on the CAM surface at specific time points, based on previous experiments with Hyp distribution in CAMs [50,51].

**Figure 3:**
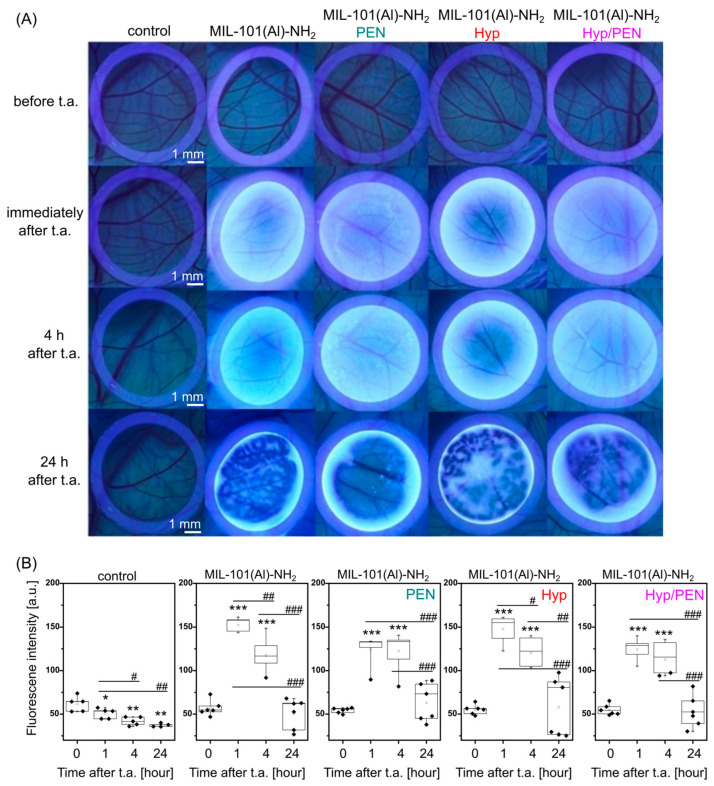
Fluorescence pharmacokinetics of MIL-101(Al)-NH_2_ and MIL-101(Al)-NH_2_ loaded with PEN, Hyp and their combination on CAM. (**A**) Fluorescence images of CAM detected in violet light (@ 405 nm) before and after administration of MOFs for 24 h. (**B**) Analysis of fluorescence intensity is determined as the average value of the image. Levels of significant difference from the control and between samples were determined using an one-way ANOVA test: * *p* < 0.05, ** *p* < 0.01, *** *p* < 0.001, # *p* < 0.05, ## *p* < 0.01 and ### *p* < 0.001.

The microvasculature of quail CAM was photographed before and after topical administration of MIL-101(Al)-NH_2_ and MIL-101(Al)-NH_2_ loaded with PEN, Hyp and the combination Hyp/PEN. The images of CAM taken in white light and at 405 nm excitation light are shown in Figure 3A. A 405 nm irradiation source was chosen to prevent deeper light penetration that could damage tissue. Therefore, superficial bacterial infection will be mostly eliminated. Furthermore, the spectral characteristics of MIL-101(Al)-NH_2_ and Hyp fall within this region, allowing excitation for bioimaging and reactive oxygen species generation to inactivate bacteria [37]. The laser power and irradiation time were set according to previous experiments on CAM [51].

A homogeneous distribution of bright emission was observed on the CAM for all MOFs analyzed. However, this distribution changed after 24 h. Analysis of the fluorescence intensities revealed a significant increase in intensities 1 and 4 h after administration (Figure 3B). In contrast, a significant decrease in intensities was observed 24 h after administration, which may be partly caused by the internalization of MOFs in CAM. It should be noted that a high biocompatibility of the MOFs and survival of the embryos was observed.

The 24 h application of MOFs to CAM can lead to localized damage to blood vessels that cannot be detected by imaging techniques but can be identified by changes in gene expression. We focused on key genes reflecting angiogenesis (vascular endothelial growth factor A (*VEGF-A)* and KDR, kinase insert domain receptor (*Quek1)*), lymphogenesis (FLT4, fms-related receptor tyrosine kinase 4 (*Quek2)*) and tissue immunostimulatory response (interferon α (*IFN-α)*, interleukin 6 (*IL-6)* and interleukin 8 (*IL-8)*).

**Figure 4:**
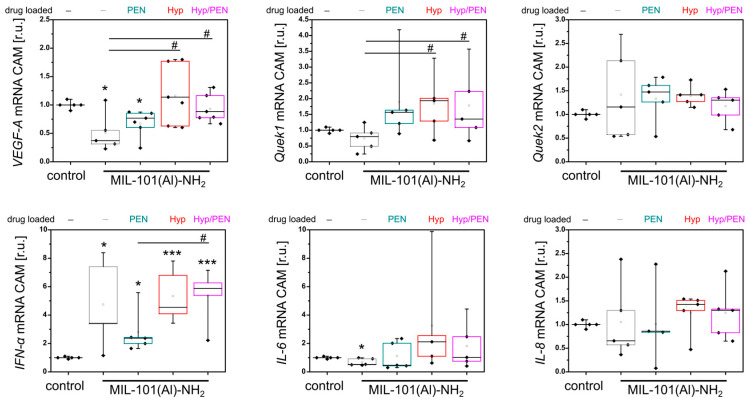
Analysis of *VEGF-A*, *Quek1*, *Quek2*, *IFN-α*, *IL-6* and *IL-8* gene expression in CAM after application of MIL-101(Al)-NH_2_ and MIL-101(Al)-NH_2_ loaded with PEN, Hyp and their combination for 24 h in the dark. Levels of significant difference from the control and between samples were determined using an one-way ANOVA test: * *p* < 0.05, *** *p* < 0.001, # *p* < 0.05.

A significant decrease in *VEGF-A* (angiogenic marker) compared to the untreated control was observed by qPCR only for MIL-101(Al)-NH_2_ and MIL-101(Al)-NH_2_ loaded with PEN (Figure 4). However, significant differences were found in *VEGF-A* and *Quek1* between CAMs treated with MIL-101(Al)-NH_2_ and those loaded with Hyp and Hyp/PEN (Figure 4). This indicates a slight impairment of angiogenesis and supports the pro-angiogenic effect of Hyp. As a result of the activity of MOFs, *IFN-α* increases significantly in all compounds analyzed (Figure 4). This result reveals an immunostimulatory effect of MIL-101(Al)-NH_2_ and its cargo.

#### 2.4.2. Interaction of MIL-101(Al)-NH_2_ and MIL-101(Al)-NH_2_ Loaded with PEN, Hyp and Their Combination with CAM of Quail Embryo Infected with *E. coli*

**Figure 5:**
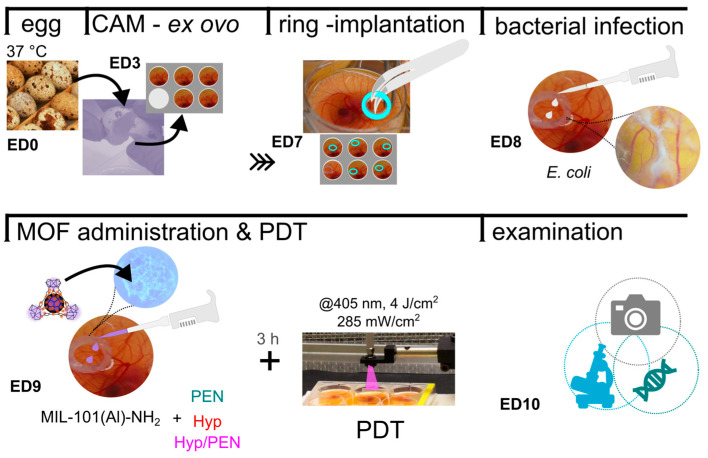
Schematic representation of CAM *E. coli* model preparation for examination.

In order to investigate the effect of MOFs on infected tissue, a CAM with bacterial infection was developed. For biosafety reasons, the *E. coli* strain was used as a model system for bacterial infection. The infected CAM was prepared and analyzed according to the protocol shown in Figure 5.

Taking into account the results obtained in Section 2.4.1. and our previous work indicating antiviral and antibacterial activity of Hyp [37], the investigation of the anti-inflammatory activity assay was performed with Hyp and MIL-101(Al)-NH_2_ loaded with Hyp and Hyp/PEN.

**Figure 6:**
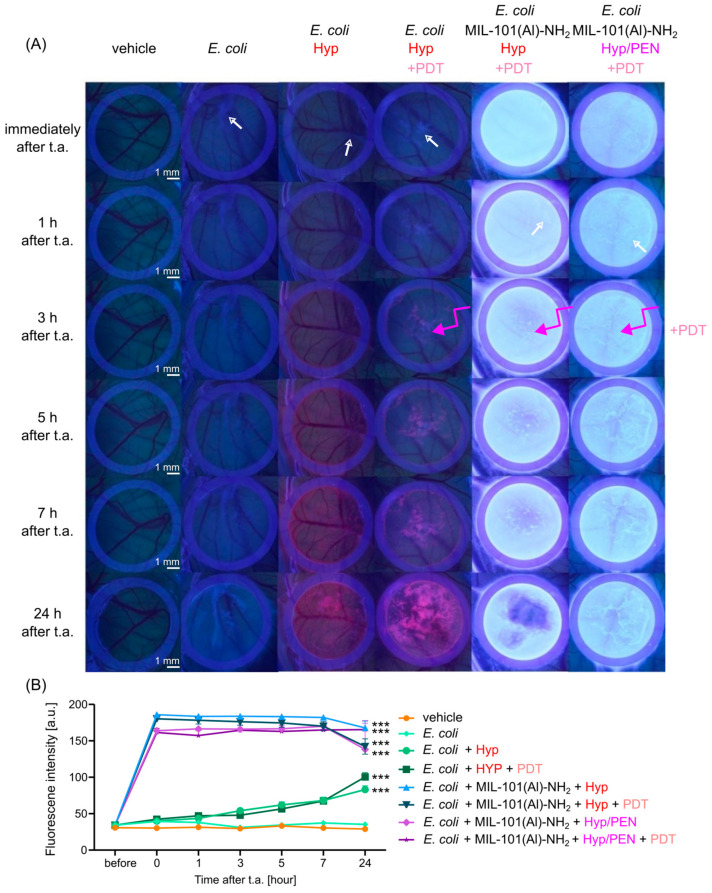
Fluorescence pharmacokinetics of Hyp and MIL-101(Al)-NH_2_ loaded with Hyp and Hyp/PEN combination on CAM infected with *E. coli* (white arrow) (**A**) Fluorescence images of CAM detected in violet light (@ 405 nm) before and after administration of MOFs for 24 h. PDT (@ 405 nm, 4 J/cm^2^) was performed 3 h (pink arrow) after topical administration (t.a.) of Hyp and MOFs. (**B**) Analysis of fluorescence intensity is determined as the average value of the image. Levels of significant difference from the control (vehicle) were determined using an one-way ANOVA test: *** *p* < 0.001.

PDT was performed 3 h after administration of the compounds to enhance the biological activity of Hyp. The fluorescence pharmacokinetics of CAM were detected in violet light at 405 nm, as shown in Figure 6A. Bacterial infection caused by endotoxins can be recognized on CAM as a whitish superficial lesion (indicated by white arrows in Figure 6A). These regions are more easily detected by Hyp (pink emission) and by a different surface profile in the presence of MOFs, which were highlighted 24 h after administration and PDT application. The increase in fluorescence intensity was confirmed by analyzing the fluorescence images (Figure 6B).

**Figure 7:**
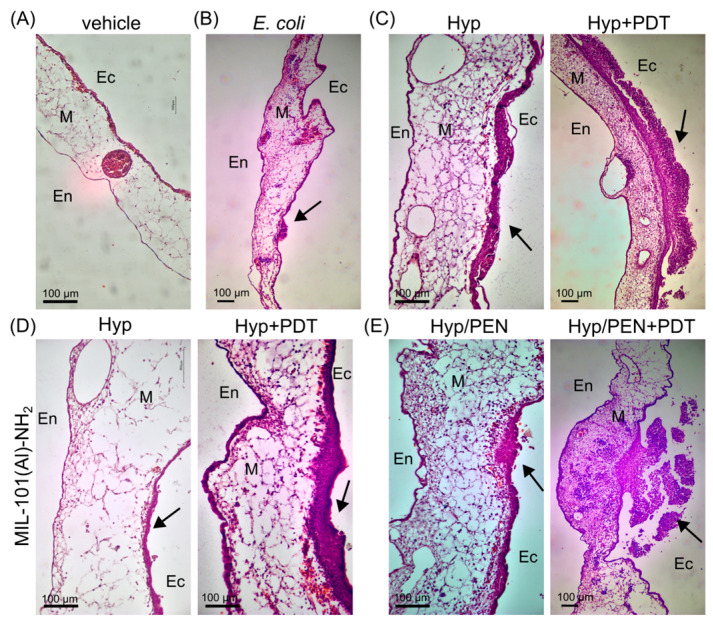
Histological sections of (**A**) CAM and (**B**) CAM infected with *E. coli* (denoted with black arrows) in the presence of (**C**) Hyp and MIL-101(Al)-NH_2_ loaded with (**D**) Hyp and (**E**) Hyp/PEN combination before and after PDT. Ec-ectoderm, En-endoderm, M-mesoderm, the black arrow indicates inoculation of *E. coli*. The tissue was labelled with hematoxylin and eosin staining.

The histological sections of CAM were stained with hematoxylin and eosin to detect malformations after *E. coli* infection and PDT. A thick layer of *E. coli* can be seen in Figure 7B–E. PDT led to a thickening of the CAM ectoderm and the bacterial layer, as indicated by the dark purple color. Molecular biological analysis was performed to understand the effect of *E. coli* and Hyp, as well as MIL-101(Al)-NH_2_ loaded with Hyp and Hyp/PEN on CAM.

**Figure 8:**
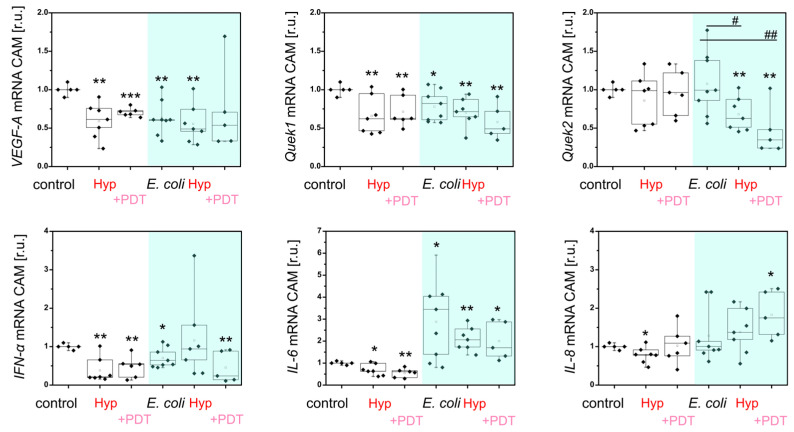
Analysis of *VEGF-A*, *Quek1*, *Quek2*, *IFN-α*, *IL-6* and *IL-8* gene expression in CAM and CAM infected with *E. coli* after application of Hyp for 24 h in the dark and with PDT (@ 405 nm, 4 J/cm^2^) 3 h after administration. Levels of significant difference from the control and between samples were determined using an one-way ANOVA test: * *p* < 0.05, ** *p* < 0.01, *** *p* < 0.001, # *p* < 0.05 and ## *p* < 0.01.

First, we analyzed Hyp and Hyp + PDT with CAM and infected CAM (Figure 8). CAM infected with *E. coli* shows decreased expression of *VEGF-A* and *Quek1* (angiogenesis) and *IFN-α* in CAM, but slight overexpression of *IL-6* (immunostimulation). Administration of Hyp and PDT resulted in a significant decrease in *VEGF-A*, *Quek1*, *IFN-α*, *IL-6* and *IL-8*. However, in infected CAM, Hyp did not affect *VEGF-A*, *Quek1* and *IFN-α*, but PDT partially decreased these genes. While *Quek2* (lymphogenesis) significantly decreased in infected CAM exposed to Hyp and PDT, *IL-6* and *IL-8* were overexpressed by these treatments (Figure 8). This suggests that Hyp may play an immunostimulatory role and affect the lymphatic system of CAM.

**Figure 9:**
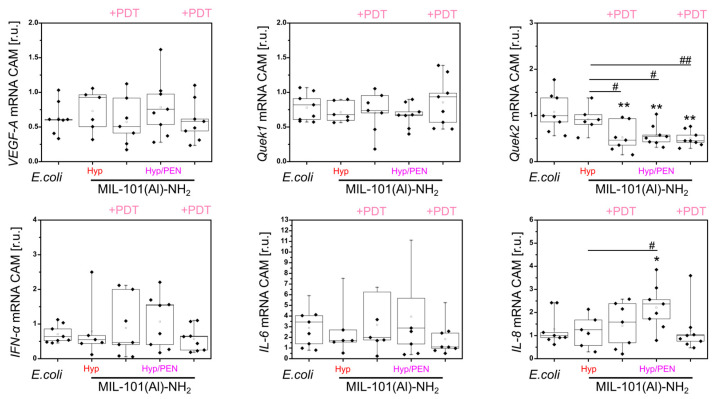
Analysis of *VEGF-A*, *Quek1*, *Quek2*, *IFN-α*, *IL-6* and *IL-8* gene expression in CAM infected with *E. coli* after application of MIL-101(Al)-NH_2_ loaded with Hyp and Hyp/PEN for 24 h in the dark and with PDT (@ 405 nm, 4 J/cm^2^) 3 h after administration. Levels of significant difference from the control and between samples were determined using an one-way ANOVA test: * *p* < 0.05, ** *p* < 0.01, # *p* < 0.05, ## *p* < 0.01.

Furthermore, MIL-101(Al)-NH_2_ loaded with Hyp and Hyp/PEN was applied to infected CAM. A significant difference was observed in the *Quek2* gene (lymphogenesis), which was decreased by MIL-101(Al)-NH_2_ loaded with Hyp + PDT and by MIL-101(Al)-NH_2_ loaded with Hyp/PEN, both in the dark and after PDT (Figure 9). In contrast, a significant increase in *IL-8* (immunostimulation) was detected in infected CAM after treatment with MIL-101(Al)-NH_2_ loaded with Hyp/PEN in the dark, while PDT suppressed this effect (Figure 9). This suggests that the combination of Hyp/PEN enhances the effect expected for Hyp and may play a role in the lymphogenesis of CAM. The weaker response of CAM tissue to MIL-101(Al)-NH_2_ loaded with Hyp compared to Hyp alone may be caused by the low concentration in the particles. A stronger effect can be ensured by increasing the Hyp concentration.

### 2.5. Antibacterial Effect of MIL-101(Al)-NH_2_ and MIL-101(Al)-NH_2_ Loaded with PEN, Hyp and Their Combination Induced by PDT

In our study, CAM infection was induced by the Gram-negative bacterium *E. coli*. Its complex outer membrane structure makes it less susceptible to PDT. Two bacterial strains studied in our research differ in cell wall structure. Gram-negative bacteria such as *E. coli* have a more complex cell wall, consisting of an inner peptidoglycan layer and an outer membrane rich in lipopolysaccharide, which is strongly negatively charged and sensitive only to certain cationic photosensitizers [52,53].

Fluorescence microscopy was employed to monitor the internalization of Hyp into bacterial cells. It was noticed that Hyp fluorescence could not be detected in *E. coli*. Consequently, we used the Gram-positive bacterium *S. epidermidis* as a model organism for PDT, since its thick peptidoglycan layer facilitates Hyp accumulation.

**Figure 10:**
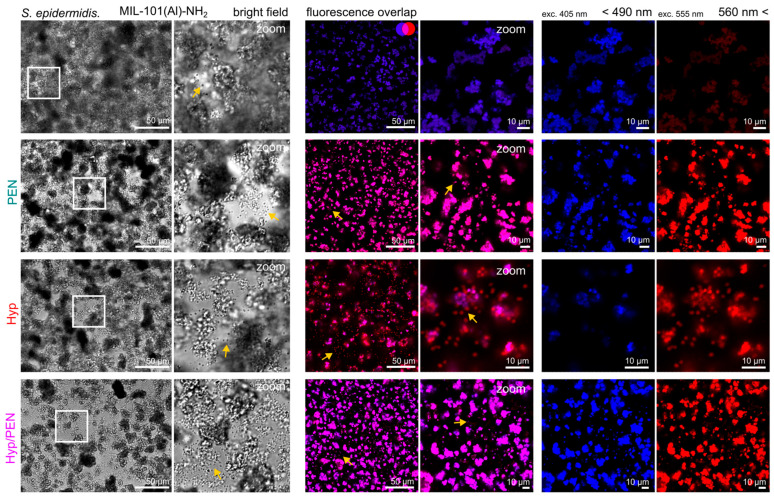
Bright field and fluorescence images of MIL-101(Al)-NH_2_ and MIL-101(Al)-NH_2_ loaded with PEN, Hyp and their combination in the presence of *S. epidermidis*. Fluorescence of the MOFs was detected in blue and red spectral regions. Hyp absorbed in bacteria is shown in red. Localization of bacteria is indicated with a yellow arrow. Overlap of blue and red signals at the same pixel position is indicated in pink. The white square indicates the region of interest, which is zoomed.

The fluorescence images of MIL-101(Al)-NH_2_ and MIL-101(Al)-NH_2_ loaded with PEN, Hyp and Hyp/PEN are shown in Figure 10. The MOFs exhibited fluorescence in both the blue and red regions of the visible spectrum. The overlap of these channels generated a pink-colored fluorescence, predominantly observed within the MOFs, appeared as dark spots in a bright field image (marked with yellow arrows). Upon incubation with Hyp-loaded MOFs, these spots were visualized in red, most prominently with MIL-101(Al)-NH_2_ loaded with Hyp, and to a lesser extent with the Hyp/PEN combination (Figure 10). The Hyp fluorescence allowed direct observation of bacterial adsorption onto MIL-101(Al)-NH_2_ particles, indicating that MOFs can act as adsorbents for Gram-positive bacteria. This effect was not observed with *E. coli*.

**Figure 11:**
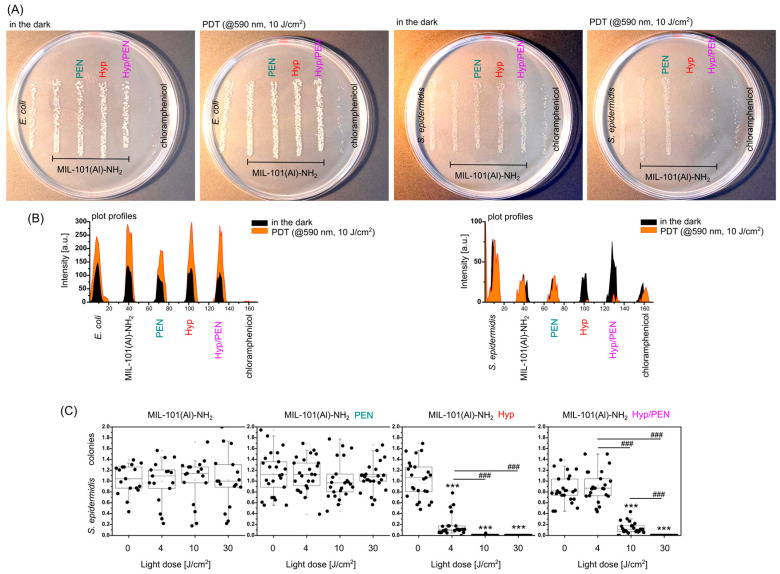
Inhibition of bacteria (*E. coli* and *S. epidermidis*) in the presence of MIL-101(Al)-NH_2_ and MIL-101(Al)-NH_2_ loaded with PEN, Hyp and their combination. (**A**) Images of bacterial colonies kept in the dark and after PDT (590 nm, 4–30 J/cm^2^). (**B**) Plot profiles of the images with colonies. (**C**) Analysis of *S. epidermidis* colonies after PDT at 0–30 J/cm^2^. Levels of significant difference from the control and between samples were determined using an one-way ANOVA test: *** *p* < 0.001, ### *p* < 0.001.

Regarding the spectral properties of Hyp and to maximize the PDT effect induced by Hyp in bacteria the irradiation source was changed to 590 nm. Bacteria were irradiated at 590 nm for different durations (4, 10, and 30 min). The bacteria were exposed to MIL-101(Al)-NH_2_ and all combinations of treatments. The results of antibacterial treatment with MOFs in the dark and after PDT are presented in Figure 11.

Representative images of bacterial colonies are shown in Figure 11A. Chloramphenicol served as a positive control for bacterial killing. While it exhibited strong antibacterial activity against *E. coli*, *S. epidermidis* displayed minor resistance to chloramphenicol at studied concentration.

In order to better recognize the differences between the treatments, the profiles of the plates with colonies were plotted in Figure 11B. In *S. epidermidis* culture treated with MIL-101(Al)-NH_2_ loaded with Hyp and Hyp/PEN, a significant decrease in the number of colonies was observed upon irradiation with light (PDT). In contrast, MIL-101(Al)-NH_2_ alone or MIL-101(Al)-NH_2_ loaded with PEN showed no differences between dark and light-exposed conditions.

The light dose dependence of *S. epidermidis* treated with MIL-101(Al)-NH_2_ loaded with Hyp and Hyp/PEN was shown in Figure 11C. There is clear evidence that the MIL-101(Al)-NH_2_ loaded with Hyp inhibits bacterial growth at 4 J/cm^2^. The same inhibitory effect was observed for Hyp/PEN at 10 J/ cm^2^. The difference is likely due to the lower active concentration of Hyp in the particles, resulting from the same nominal particle weights. These results also demonstrate that PDT induced by Hyp can overcome the resistance of *S. epidermidis* to PEN, producing a stronger antibacterial effect than chloramphenicol.

## 3. Discussion

Bacterial infections are an increasingly serious global health concern [54]. The emergence of resistance to multiple antibiotics is driving the search for alternative therapeutic strategies. PDT is one of the most promising therapeutic approaches to reduce bacterial and microbial infections non-invasively [55]. It exerts a dual effect: inhibiting bacterial growth via light-induced ROS generation while simultaneously stimulating the immune system to mount a rapid defense.

The present work exploits the advantages offered by MOFs, the modern and intelligent mesoporous materials with multimodal applications [56]. MIL-101(Al)-NH_2_ proved to be a very effective transport system for the photosensitizer—Hyp and its co-delivery with PEN in this study. This system is suitable for bioimaging due to the bright emission of blue light. Dong and co-workers presented the ability to control the emission of MIL-101(AL)-NH_2_ by interacting with amino acids, and therefore can be used as a sensor for lysine, arginine and histidine [57]. Song and co-workers improved this sensor by incorporating eosin Y into the MIL-101(Al)-NH_2_ to obtain a dual sensor with blue and yellow emission [58]. We achieved dual sensing in the blue and orange regions by adsorbing Hyp into the MIL-101(Al)-NH_2_ pores.

To illustrate the bioimaging applicability of MIL-101(Al)-NH_2,_ we used a preclinical CAM model. This model is of great advantage, because it complies with the 3R (Replacement, Reduction, and Refinement) policy for the use of animals in research. In this context, we have developed an in vivo model of bacterial infection on CAM that can be used in multiple replicates with simple manipulation. Moreover, the CAM system provided a dynamic in vivo platform to evaluate the angiogenic, inflammatory, and antibacterial responses induced by the hybrid treatment under controlled conditions. Its rich vascularization enabled real-time visualization of vascular activation and immune cell recruitment, validating the immunostimulatory role of MIL-101(Al)-NH_2_ and its potential to enhance PDT efficiency against *E. coli*. Although differences exist between avian and mammalian immunity, the CAM model therefore represents a valuable intermediate step for assessing PDT-induced immunomodulation prior to mammalian studies.

Using the CAM model, we demonstrated that MIL-101(Al)-NH_2_ stimulates the immune response within 24 h of topical administration, as evidenced by increased *IFN-α* levels that support antimicrobial defense. When loaded with Hyp, these MOFs enhanced angiogenic factors in the CAM, including *VEGF-A* and *Quek1*. This may indicate the restoration of blood vessels damaged by the MOFs, which were probably suppressed by MIL-101(Al)-NH_2_ due to the local pressure of the particles. Several papers have reported the pro-angiogenic effect of MOFs, which promotes healing processes and tissue acceptance of the composites [59,60]. This supports the hypothesis that MIL-101(Al)-NH_2_ can promote tissue regeneration when applied to CAM.

Infected CAMs were exposed to MIL-101(Al)-NH_2_ Hyp and Hyp/PEN, which were expected to have immunostimulatory effects on damaged CAMs. This infection resulted in an increase in *IL-6* and a non-significant increase in *IFN-α* and a significant increase in *IL-8* (key mediators in the immune system) after combined Hyp/PEN treatment, but this was reversed by PDT. *Quek2* was significantly decreased by MIL-101(Al)-NH_2_ Hyp and Hyp/PEN treatment, suggesting suppression of lymphogenesis. This effect could be caused by a combination of *E. coli* and MOFs applied on the CAM surface, as the lymphatic system in the CAM is intertwined with the vasculature [61].

**Figure 12:**
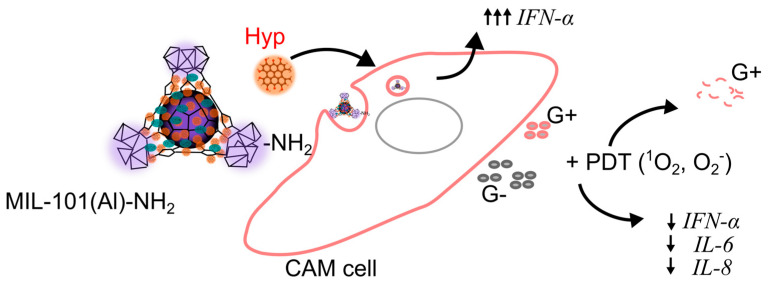
Schematic representation of MIL-101(Al)-NH_2_ and Hyp mechanism on immunostimulation in CAM and PDT effect on bacteria (Gram-positive (G+), Gram-negative (G−)).

To summarize, prepared MOFs inhibited angiogenesis in the dark and had an immunostimulation effect (Table 1). The hypothesis of MOFs effects on immunostimulatory mechanism is shown in Figure 12. In contrast, Hyp-MOFs induced PDT attacked lymphogenesis of *E. coli* infected CAM (Table 1), probably due to deeper penetration of Hyp into endothelium.

The immunostimulatory effect of MOFs is a key property that enhances their ability to combat infection. Chen and co-workers found that MIL-101(Al) capped with palmitic acid enhances the immune response and can be used as a nano-adjuvant to treat pathogens [62]. The combination of MIL-101(Al)-NH_2_ and Hyp proved to be not only effective against bacteria, but also promising in antiviral and anticancer treatment [37].

According to our findings, the antibacterial activity of MIL-101(Al)-NH_2_ loaded with Hyp or Hyp/PEN was dependent on both concentration and light dose.

Hyp-mediated PDT generates ROS such as ^1^O_2_ and superoxide radicals upon photoactivation. These inflict oxidative damage on multiple bacterial targets in a manner largely unaffected by conventional resistance determinants such as β-lactamases, efflux pumps, or altered penicillin-binding proteins [63,64]. Importantly, we observed that our co-delivery system was able to overcome PEN resistance in *S. epidermidis*. We propose that, in the presence of the MOF carrier, PEN is delivered simultaneously or sequentially in close proximity to the bacterial target at the moment when the cell envelope is compromised by PDT-generated ROS. Under these conditions, the antibiotic can more readily reach its PEN-binding protein targets, bypassing the usual diffusion, permeability, or enzyme-mediated resistance constraints. Consequently, the combined modality can effectively re-sensitize a previously resistant strain to the β-lactam mechanism of action. In addition, our MOFs not only serve as a co-delivery platform for Hyp and PEN but may also create a microenvironment that locally concentrates the photosensitizer and antibiotic at the bacterial surface, enhancing damage and uptake. This interpretation is supported by our fluorescence microscopy data, which show that MIL-101(Al)-NH_2_ particles can act as effective adsorbents for Gram-positive bacteria, thereby increasing the degree of physical contact between bacteria and the delivered compounds.

PDT, however, proved to be an important parameter for inhibiting bacterial growth and was more successful than the standard antibiotics PEN and chloramphenicol. While our antibacterial PDT was successful against Gram-positive *S. epidermidis*, it showed limited activity against Gram-negative species such as *E. coli*. Future optimization, for example, by adjusting particle morphology, could enhance efficacy against Gram-negative bacteria. Zhao and co-workers were able to target *E. coli* with MIL-101(Al)-NH_2_, which was altered in the morphology of spiny particles [65]. Future surface engineering of MOFs could enhance their affinity for the lipophilic surfaces characteristic of Gram-negative bacteria, potentially improving antibacterial efficacy.

In conclusion, we have shown that MOF materials have promising potential for antibacterial treatment through immunostimulatory effect and inhibition of bacterial growth by PDT. MIL-101(Al)-NH_2_ as drug carriers proved to be highly biocompatible, which makes them suitable for bioimplantation applications. In addition, their antibacterial potential makes them very promising to reduce infections of implants, as reported for orthopedic implants and the regulation of their infections [66]. Remarkably, MOFs can be tailored with functional groups to achieve more precise particle targeting. The next step may be to modify the MOFs to improve their interaction with the Gram-negative bacterial wall and to replace Hyp with another photosensitizer, preferably in a cationic form. Therefore, the potential and broad applicability of this material goes beyond our work, but it is a great challenge for further studies.

## 4. Materials and Methods

### 4.1. Synthesis and Characterization of MIL-101(Al)-NH_2_ and MIL-101(Al)-NH_2_ Loaded with PEN, Hyp and Their Combination

#### 4.1.1. Synthesis of MIL-101(Al)-NH_2_ and MIL-101(Al)-NH_2_ Loaded with PEN, Hyp and Their Combination

MIL-101(Al)-NH_2_ matrices were synthesized according to the protocol described in our previous work [37]. The loading of PEN, Hyp (abcam, Cambridge, UK), and their combinations into the MIL-101(Al)-NH_2_ framework was carried out by the impregnation method under ambient conditions. Specifically, 500 mg of MIL-101(Al)-NH_2_ was impregnated with 50 mg of PEN dissolved in 5 mL of deionized water (equivalent to 100 mg of PEN per 1 g of MIL-101(Al)-NH_2_; designated as MIL-101(Al)-NH_2_ + PEN), 0.1 mg of Hyp dissolved in 5 mL of methanol (equivalent to 0.2 mg of Hyp per 1 g of MIL-101(Al)-NH_2_; designated as MIL-101(Al)-NH_2_ + Hyp), or a mixture of 50 mg PEN (in water, 2.5 mL) and 0.1 mg Hyp (in methanol, 2.5 mL) (equivalent to 100 mg of PEN and 0.2 mg of Hyp per 1 g of MIL-101(Al)-NH_2_; designated as MIL-101(Al)-NH_2_ + PEN + Hyp), corresponding to a weight ratio of MOF:PEN:Hyp = 1:0.10:0.0002. After the impregnation step, the samples were evaporated ensuring that the entire amount of the drug was loaded into the sample. Evaporation took approximately 8 h at 60 °C. The samples were then dried under vacuum at room temperature to remove any residual moisture and remaining solvent traces. The drug-loaded MIL-101(Al)-NH_2_ composites were stored in tightly sealed glass vials at 4 °C in the dark to prevent degradation of the bioactive compounds before further characterization, release studies and biological tests.

#### 4.1.2. Characterization of MIL-101(Al)-NH_2_ and MIL-101(Al)-NH_2_ Loaded with PEN, Hyp and Their Combination

IR spectra were recorded on a Nicolet 6700 (Thermo Scientific, Waltham, MA, USA) using the ATR (diamond) mode. Each spectrum represents the average of 128 scans collected in the range 4000–400 cm^−1^ with a resolution of 2 cm^−1^. Samples were dried under vacuum prior to analysis to minimize residual moisture/solvent contributions.

PXRD patterns were acquired on a D2 PHASER diffractometer (Brucker, Karlsruhe, Germany) using Cu Kα, *λ* = 1.5406 Å radiation, operated at 40 kV and 40 mA. Data were collected over 2*θ* range of 10–50° with a step size of 0.02° and a counting time of 2 s per step in Bragg–Brentano geometry. Instrumental broadening was determined using a LaB_6_ standard. Measured patterns were compared with the simulated diffractogram generated from the crystallographic model of MIL-101(Cr)-NH_2_ [41] to verify phase purity and structural integrity after loading.

Nitrogen physisorption analysis was conducted to assess the porous characteristics of the prepared samples, following the procedure outlined in [67,68]. N_2_ adsorption–desorption isotherms at 77 K were measured on an ASAP 2020 (Micromeritics, Norcross, GA, USA) surface area analyzer. Before measurement, the pristine MIL-101(Al)-NH_2_ sample was outgassed under dynamic vacuum at 100 °C for 12 h, while drug-loaded composites were degassed at 80 °C for 12 h to prevent possible thermal degradation of the encapsulated molecules. The specific surface area (*S_BET_*) was calculated from the adsorption branch using the BET method within the linear relative pressure range selected according to the Rouquerol criteria (typically *p*/*p*_0_ ≈ 0.05–0.30) [69]. The total pore volume (*V_p_*) and pore size (*d*) were derived from the adsorption branch using a Non-Local Density Functional Theory (NLDFT) adsorption kernel.

Thermogravimetric analysis (TGA) measurements were performed on a SetsysEvolution instrument (Setaram, Caluire, France) under an air flow (80% Ar and 20% O_2_) of 40 mL·min^−1^. Approximately 10 mg of sample was heated from 30 to 900 °C (with an isothermal step at 80 °C for 30 min) at a rate of 10 °C·min^−1^ in corundum pans.

#### 4.1.3. PEN and Hyp Release Kinetics from MIL-101(Al)-NH_2_

Release experiments of PEN and Hyp were carried out simultaneously in 40 mL phosphate-buffered saline (PBS, pH 7.4) at 37 °C. 10 mg of the carrier with loaded drug (MIL-101(Al)-NH_2_ + Hyp, MIL-101(Al)-NH_2_ + PEN or MIL-101(Al)-NH_2_ + Hyp/PEN) was weighed and dispersed in the saline solution. After 1 h, 10 mL of fetal bovine serum (FBS, Biosera, Cholet, France) was added to the medium. The release study was conducted over a 24 h period and was performed in triplicate.

Aliquots (3 mL) were withdrawn at predetermined time intervals of 0.25, 0.5, 1, 2, 3, 4, 5, 6, and 24 h. Each withdrawn fraction was analyzed for absorbance and fluorescence. UV–Vis absorption spectra were recorded using a Jasco spectrophotometer (Kyoto, Japan), and fluorescence spectra were measured with a Jasco FP-8550 spectrofluorometer (Kyoto, Japan). Samples were excited at 555 nm (for Hyp). After measurement, the aliquots were returned to the release medium to maintain constant volume.

For quantitative determination of PEN, 0.2 mL of the release medium was centrifuged, and 0.1 mL of the supernatant was used for HPLC analysis. Chromatographic separation was performed on a reversed-phase C18 column (250 mm × 4.6 mm, 5 μm particle size) maintained at 30 °C. The mobile phase consisted of acetonitrile/water (typically 30:70, *v*/*v*) with a flow rate of 1.0 mL·min^−1^ under ~1000 psi. Detection was carried out with a UV detector set at 254 nm, which is commonly applied for PEN derivatives.

### 4.2. Interaction of MIL-101(Al)-NH_2_ and MIL-101(Al)-NH_2_ Loaded with PEN, Hyp and Their Combination with Quail Chorioallantoic Membrane Model (CAM)

#### 4.2.1. Preparation of Quail CAM In Vivo Model

Fertilized Japanese quail (Coturnix japonica) eggs were incubated with egg rotation turned off (Bios Midi, Sedlčany, Czech Republic) at a temperature of 37 °C and a relative humidity of 50–60%. On ED3 (embryonic day), the eggs were sterilely (surface disinfection with 70% EtOH) tipped into 6-well culture plates (Sarstedt, Nümbrecht, Germany) using scissors in a laminar flow box. After preparing the ex ovo culture, the eggs were placed in an incubator under the same conditions until the day of the experiment.

#### 4.2.2. Biocompatibility of MIL-101(Al)-NH_2_ and MIL-101(Al)-NH_2_ Loaded with PEN, Hyp and Their Combination with Quail CAM In Vivo Model and *E. coli* Infected CAM Model

For the experiment, an ex ovo CAM model of quail embryos was used. The nanoparticles were tested at a concentration of 2 mg/mL. On ED8, CAMs were applied with—PBS (phosphate buffer) as a control group (*n* = 10), 2 mg/mL MIL-101(Al)–NH_2_ (*n* = 13), 2 mg/mL MIL-101(Al)–NH_2_ PEN (*n* = 11), 2 mg/mL MIL-101(Al)–NH_2_ Hyp (*n* = 12), 2 mg/mL MIL-101(Al)–NH_2_ Hyp/PEN (*n* = 11). Individual solutions were applied to the CAM surface in silicone rings (∅ 10 mm) in a volume of 80 µL. The areas in the rings were photographed before application, immediately after, 4 h and 24 h after application. They were photographed in fluorescent light (LED light source, 405 nm) at all time intervals and in white light (Canon 6D II, MP-E 65 mm f/2.8 Macro lens, Tokyo, Japan), only before application and 24 h after topical application. The samples were taken at ED9 (24 h after the administration) for histological and molecular processing. During the experiment, embryo survival was recorded. All procedures were performed at low light intensity.

The standard strain of Gram-negative bacteria *E. coli* DH5α was inoculated into liquid LB medium (1% peptone, 0.5% yeast autolysate, 1% NaCl, pH 7–7.2) and diluted to a working concentration of 1.08 × 10^8^ cells/mL medium. For the experiment, we used an ex ovo CAM model of quail embryos. On ED8, CAM were applied with—0.1% DMSO (in PBS) (control) (*n* = 9), LB medium (*n* = 8), LB medium + HYP (*n* = 10), LB medium + HYP + PDT (*n* = 10), *E. coli* (*n* = 12), *E. coli* + HYP (*n* = 10), *E. coli* + HYP + PDT (*n* = 8), *E. coli* + 2 mg/mL MIL-101(Al)–NH_2_ Hyp (*n* = 9), *E. coli* + 2 mg/mL MIL-101(Al)–NH_2_ Hyp + PDT (*n* = 10), *E. coli* + 2 mg/mL MIL-101(Al)–NH_2_ Hyp/PEN (*n* = 11), *E. coli* + 2 mg/mL MIL-101(Al)–NH_2_ Hyp/PEN (*n* = 11). On ED8, we added LB medium and *E. coli* in LB medium in a volume of 80 µL to silicone rings (∅ 10 mm) on the CAM surface and cultured the embryos for another 24 h (until ED9); see Figure 5. On ED9, we photographed the CAM in the area of the silicone rings in white and fluorescent light. Subsequently, 0.1% DMSO was added to the control group, to the medium and *E. coli* in the medium. Further Hyp (Sigma-Aldrich, Bratislava, Slovakia) was applied in a concentration of 2 µg/g embryo weight (79 μM solution in PBS, the DMSO solvent content was 0.1%), 2 mg/mL MIL-101(Al)–NH_2_ Hyp and 2 mg/mL MIL-101(Al)–NH_2_ Hyp/PEN. All in a volume of 80 µL.

#### 4.2.3. Fluorescence Pharmacokinetics of MIL-101(Al)-NH_2_ and Hyp in CAM Models

From CAM photographs of the silicone rings in the fluorescent LED light with a wavelength of 405 nm, the fluorescence intensities of the applied fluorescent substances over time (MOFs or Hyp) were analyzed using ImageJ software 1.48v [70]. Using the program, the photograph was divided into RGB color spectra (red, green, blue). The red spectrum was used for analysis. The marked area in the ring was the same for all photographs in a given experiment.

#### 4.2.4. Photodynamic Treatment of CAM Models Induced by Combination of MIL-101(Al)-NH_2_, PEN and Hyp

At 3 h after Hyp application, the area inside the rings was treated with a diode laser for 2 min (405 nm, 100 mV, 285 mW/cm^2^, 4 J/cm^2^). The areas were photographed immediately after Hyp and MOF particle administration (0 h) and then 1, 3, 5, and 7 h after application under fluorescent light (LED light, 405 nm) and 24 h after application under fluorescent and white light. At ED10 (24 h after Hyp and MOF application), after photographing under fluorescent and white light, we collected the samples for histological and molecular processing.

#### 4.2.5. Histological Evaluation of Treated CAM Models

For histological analysis, CAM was fixed with 4% paraformaldehyde (PFA) solution. Subsequently, the tissue was dehydrated in ethanol and soaked in paraffin. After 24 h, the samples were overlaid with Paraplast Plus for tissue embedding in casting molds. 5 µm sections from three parts of the sample were cut on a hand microtome (Nahita 508, Auxilab, Navarra, Spain) and stained with hematoxylin–eosin.

#### 4.2.6. Evaluation of Molecular Biology Parameters in CAM Models

Total RNA was isolated from the samples using TRI-Reagent (Molecular Research Center, Cincinnati, OH, USA) according to the protocol of Chomczynski and Sacchi [71]. 1 mL of TRI-Reagent was added to approximately 50 mg of frozen tissue, followed by homogenization on ice on an UltraTurrax T18 homogenizer (IKA-Labortechnik, Staufen, Germany), and phase separation was performed with chloroform. RNA was precipitated with freeze-dried isopropanol, washed with 75% ethanol, and dissolved in 50 µL RNAse-free water.

Prior to transcription of the isolated RNA, its concentration and purity were measured using the spectrophotometer Multiskan™ GO Microplate (Thermo Fisher Scientific, Bremen, Germany). Possible genomic DNA contaminations were removed using the RapidOut DNA Removal Kit. According to protocol, 2 µg purified RNA was used for transcription into cDNA on an RT cycler (PTC-150, MJ Research, Waltham, MA, USA). The reaction mixture contained: oligo (dT)18 primer (100 µM), random hexamer primer (100 µM), RT buffer 5× (250 mM Tris-HCl pH 8.3, 375 mM KCl, 15 mM MgCl_2_ with 0.1 M DTT), Ribo LockRNase Inhibitor (40 U/µL), DNTP Mix (10 mM), and RevertAid Reverse Transcriptase (200 U/µL). Final cDNA was 10× diluted and stored at −80 °C.

Quantitative PCR (qPCR) was performed to determine the expression of IL-6 (interleukin 6), IL-8 (interleukin 8), IFN-α (interferon α), VEGF-A (vascular endothelial growth factor A), Quek1 (KDR, kinase insert domain receptor) and Quek2 (FLT4, fms-related receptor tyrosine kinase 4) genes. Two housekeeping genes were used in the reaction, β-actin (ACTB, actin beta) and GADPH (glyceraldehyde 3-phosphate dehydrogenase). The primers (Table 2) were synthesized according to previously tested sequences [72,73].

In the PCR reaction, FastStart DNA Master SYBR Green I (Roche, Mannheim, Germany) was used, and the final primer concentration was 0.5 µM. The reaction mixture per sample consisted of 3 µL water, 2 µL PrimerMix (5 µM), 10 µL MasterMix 2× (SYBR Green), and 5 µL 10× diluted cDNA. Reactions were run on the cycler LightCycler^®^ Nano (Roche, Basel, Switzerland) and subsequently, the signal intensity during amplification was detected using the software LightCycler^®^ Nano SW1.1. The first step consists of activating DNA polymerases and denaturing double-stranded DNA molecules at 95 °C for 10 m following 42 cycles, which consist of the following steps: denaturation (15 s, 94 °C), annealing (20 s, 55 °C), and elongation (20 s, 72 °C). The program ended at 95 °C in 10 m.

### 4.3. Interaction of MIL-101(Al)-NH_2_ and MIL-101(Al)-NH_2_ Loaded with PEN, Hyp and Their Combination with Bacterial Strains

The bacterial strains *Staphylococcus epidermidis* CCM 4418 (*S. epidermidis*) and *Escherichia coli* CCM 3954 (*E. coli*) were purchased from the Czech Collection of Microorganisms (Masaryk University, Brno, Czech Republic). The bacteria were cultured overnight at 37 °C and 250 rpm in Mueller–Hinton broth (ThermoFisher Scientific, Waltham, MA, USA). After dilution of the colonies in Mueller–Hinton broth, the optical density was adjusted to 0.2 at 600 nm. For each strain, 2 mL samples were prepared in which the particles were dispersed at a final concentration of 1 mg/mL: MIL-101(Al)–NH_2_, MIL-101(Al)–NH_2_ loaded with PEN, MIL-101(Al)–NH_2_ loaded with Hyp and MIL-101(Al)–NH_2_ loaded with Hyp/PEN, which were kept in the dark at 37 °C and 250 rpm for 2 h and then irradiated (@ 590 nm LED light, light doses: 4, 10 and 30 J/cm^2^). The negative control sample contained no nanoparticles. The positive control contained chloramphenicol (Sigma-Aldrich, St. Louis, MO, USA). Spots of 10 µL were applied to the surface of Mueller–Hinton agar plates, which were then tilted to allow the liquid to run in strips before drying under sterile conditions. The plates were incubated overnight at 37 °C. All PDT experiments were conducted under low light intensity to prevent premature Hyp photoactivation. Images of the Petri dishes containing the bacterial colonies were captured using a Canon EOS R7 camera (Canon, Tokyo, Japan). Colony counts and profile plots from selected plates were determined using ImageJ software.

Fluorescence images of MOFs and bacteria incubated for 2 h were captured using a confocal fluorescence microscope system (LSM 700, Zeiss, Oberkochen, Germany). The samples were excited with solid-state lasers at 405 nm and 555 nm. Emission was detected <490 nm and above 560 nm. The bright field and fluorescence images were analyzed using ImageJ software.

## Figures and Tables

**Table 1:** Variation in angiogenic, lymphatic and immunostimulatory related genes in quail CAM (↑ upregulation, ↓ downregulation, * compared to *E. coli* infected CAM).

		MIL-101(Al)-NH_2_			
Effect of treatment on:	*E. coli*	-	+ PEN	+ Hyp	+ Hyp/PEN
				* E. coli * PDT	* E. coli * PDT
*angiogenesis*					
*VEGF-A*	↓↓	↓	↓	↑ — —	— — —
*Quek1*	↓	—	—	— — —	— — —
*Lymphogenesis*					
*Quek2*	—	—	—	— — ↓↓	— ↓↓ ↓↓
Immunostimulation					
*IFN-* *α*	↓	↑	↑	↑↑↑ — —	↑↑↑ — —
*IL-6*	↑	↓	—	— — —	— — —
*IL-8*	—	—	—	— — —	— ↑ —

**Table 2:** Primers used for qPCR for amplification of genes (*Tm*-melting temperature).

Gen	Primers (5′-3′)	Tm (°C)
*IL-6*	GGTGATAAATCCCGATGAAGT TCTCCATAAACGAAGTAAAGTCTC	61.5 59.4
*IL-8*	CTGAGGTGCCAGTGCATTAG AGCACACCTCTCTTCCATCC	63.5 63.3
*IFN*-*α*	CCTTGCTCCTTCAACGACA CGCTGAGGATACTGAAGAGGT	64.1 62.3
*VEGF-A*	CGGAAGCCCAATGAAGTTATC GCACATCTCATCAGAGGCACA	59.4 64.0
*Quek1*	CATCAATGCGAATCATACAGTTAAG CATTCACAAGCAGGGTGAATG	60.9 59.4
*Quek2*	GAGATGAGCGGCTGATCTACTTC GAAAGGTTCAGGCGATACCAC	64.7 61.3
β-*aktin*	TGAACCCCAAAGCCAACAG CCACAGGACTCCATACCCAAG	66.0 65.5
*GAPDH*	GAACGCCATCACTATCTTCCAG GGGCTGAGATGATAACACGC	62.1 60.5

## Data Availability

Data available upon request.
